# *Akkermansia muciniphila*: a potential candidate for ameliorating metabolic diseases

**DOI:** 10.3389/fimmu.2024.1370658

**Published:** 2024-03-20

**Authors:** Huifang Niu, Minfeng Zhou, Daniel Zogona, Zheng Xing, Ting Wu, Rui Chen, Dandan Cui, Fengxia Liang, Xiaoyun Xu

**Affiliations:** ^1^ Key Laboratory of Environment Correlative Dietology (Ministry of Education), Hubei Key Laboratory of Fruit Vegetable Processing Quality Control (Huazhong Agricultural University), School of Food Science and Technology, Huazhong Agricultural University, Wuhan, Hubei, China; ^2^ Union Hospital Affiliated to Tongji Medical College, Huazhong University of Science and Technology, Wuhan, Hubei, China; ^3^ School of Acupuncture and Bone Injury, Hubei University of Chinese Medicine, Wuhan, China

**Keywords:** *Akkermansia muciniphila*, metabolic diseases, obesity, gut microbiota, health

## Abstract

Metabolic diseases are comprehensive disease based on obesity. Numerous cumulative studies have shown a certain correlation between the fluctuating abundance of *Akkermansia muciniphila* and the occurrence of metabolic diseases. *A. muciniphila*, a potential probiotic candidate colonized in the human intestinal mucus layer, and its derivatives have various physiological functions, including treating metabolic disorders and maintaining human health. This review systematically explicates the abundance change rules of *A. muciniphila* in metabolic diseases. It also details the high efficacy and specific molecules mechanism of *A. muciniphila* and its derivatives in treating obesity, type 2 diabetes mellitus, cardiovascular disease, and non-alcoholic fatty liver disease.

## 1 Introduction

**Figure 1:**
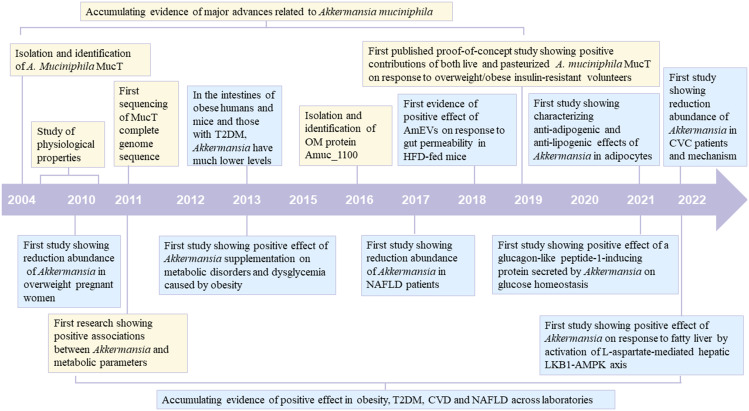
Timeline of major advances related to *Akkermansia muciniphila* and its positive effect on metabolic diseases (Obesity, T2DM, CVD and NAFLD). The blue box indicates major advances related to *A. muciniphila*. The yellow box indicates its positive effect on metabolic diseases T2DM, type 2 diabetes; CVD, cardiovascular disease; NAFLD, nonalcoholic fatty liver disease.

The gut microbiota is recognized as one of the pivotal environmental factors in regulating host health. The compositional imbalance of intestinal microbiota is associated with the emergence of various diseases. Multiple studies have reported a significant correlation between metabolic diseases, including obesity (1), type 2 diabetes (T2DM) (2), cardiovascular disease (CVD) (3), and nonalcoholic fatty liver disease (NAFLD) (4) and a specific bacterium. In the process of observing different bacteria, *Akkermansia muciniphila* has been repeatedly mentioned as a potential candidate due to its unique function, high prevalence, and abundance in nearly all life stages. However, products containing *A. muciniphila* are unavailable in various countries. The exact mechanism underlying *A. muciniphila*, exerting a probiotic effect on the host, is not fully understood. In this manuscript, we reviewed the history of this microbe from its discovery to the first investigations associating *A. muciniphila* with metabolic diseases (**Figure 1**). We systematically elaborated on the significant role played by this bacterium in metabolic diseases and its mechanism of action through a review of current human and animal experiments.

## 2 Characteristics of *A. muciniphila*

*A. muciniphila*, a number of the phylum Verrucomicrobia, is an oval-shaped, non-motile, strictly anaerobic Gram-negative strain. Since its isolation in 2004, the number of available studies about *A. muciniphila* has increased exponentially. *A. muciniphila* can be cultivated in the following media: brain heart soak, porcine gastric mucin medium, Columbia broth, trypsin soy, and synthetic medium (16 g/L soy protease, 4 g/L threonine, 25 mM glucose, and 25 mM N-acetylglucosamine). It has an optimal growth temperature of 37°C and an optimal pH of 6.5, and it must be cultured in either 100% N_2_ or 5% H_2_, 10% CO_2_, 85% N_2_ (5–8). *A. muciniphila* is highly abundant in the intestinal tract, accounting for approximately 1% to 4% of the total intestinal microbiota. *A. muciniphila* uses mucin as its sole carbon and nitrogen source, giving it a distinct survival advantage that is not strictly diet-dependent. *In vitro*, it preferentially utilizes monosaccharides, such as glucose and fructose, over N- acetylglucosamine and N-acetylgalactosamine to grow (9). The whole genome sequencing of MucT further confirmed that *A. muciniphila* primarily depends on a series of hydrolytic enzymes to degrade mucins, including proteases, sulfatases, *β*-N acetylhexosaminidase, glycosyl-hydrolases enzymes and sialidases.

*A.muciniphila* is highly prevalent and abundant in humans and animals. It primarily colonizes the outer mucus layer of the gastrointestinal tract, and its distribution in the intestinal tract was uneven, with the highest abundance in the cecum. *A. muciniphila* was also detected in rats, horses, rock thunderbirds, otters, dairy calves, guinea pigs, Burmese pythons, and zebrafish (10–16). In addition, *A. muciniphila* was found to be enriched in human breast milk, utilizing oligosaccharides in human breast milk as a carbon source (17), indicating that it has the potential for vertical transmission from mother to child. A large-scale population genomic analysis of the genus *A. muciniphila* using 88 isolated genomes and 2226 genomes showed that the human gut contains five candidate species of *A. muciniphila* (18). Their 16S rRNA gene sequences were surprisingly similar, but there were significant genome-wide differences. *A. muciniphila* strains from 22 Chinese intestines were isolated and identified by genomic fingerprinting (Enterobacterial Repetitive Intergenic Consensus Polymerase Chain Reaction), which unexpectedly indicated that they could be classified into 12 subclasses (19). Based on 39 isolates, an evolutionary tree was constructed using Single Nucleotide Polymorphism loci of core genes, and *A. muciniphila* was divided into three subgroups, AmI, AmII and AmIII, with significant variations in KEGG and GO functional annotations (20).

The functional activity of *A. muciniphila* as a potential candidate strain for alleviating metabolic diseases depends on its abundance level. Multiple factors affect the abundance of *A. muciniphila* in the host’s intestinal tract. Age is the first factor. Initially, vertical transmission of *A. muciniphila* to infants occurs by human breast milk, with one- month-old infants containing 2.05 to 4.36 log cells/g of feces. The number of *A. muciniphila* increases rapidly with age, nearly doubling in infants aged 6 to 12 months. By two years of age, a complete mucin-degrading microbiota is established, approaching the number found in adults. In addition, *A. muciniphila* concentrations are significantly lower in fecal samples of the elderly compared to adults (21). Diet is the second factor. Long-term consumption of high-sugar or high-fat diets alters the structure and composition of the intestinal microbiota and reduces the amount of *A. muciniphila* (22), whereas a restrictive diet has the opposite effect (23). It is the cause of the increased abundance of *A. muciniphila* in postoperative patients after gastric-bypass surgery (24). Similar to gastric bypass surgery, calorie restriction also positively influences the gut microbiota and the abundance of *A. muciniphila*. Recent studies indicate that intermittent fasting enhances the levels of *Lactobacillus* and *Bifidobacterium* in healthy mice while diminishing *Helicobacter*, *Prevotella*, and *Bacteroides* populations (25–27). Following 28 days of intermittent fasting treatment, db/db mice demonstrated a marked rise in *Lactobacillus* levels and the production of butyrate-producing *Odoribacter* in their intestines (28). Additionally, enrichment of *A. muciniphila* is frequently observed in individuals or animals undergoing calorie restriction (29–33). Changes in the gut microbiota due to calorie restriction ultimately result in increased levels of short-chain fatty acids (34). These fatty acids, upon binding to their respective G protein-coupled receptors, stimulate the production of peptide YY (PYY), thereby contributing to appetite suppression, decreased gastrointestinal motility, and reduced calorie intake. Supplementation with prebiotics, fructo- oligosaccharides, FODMAP (fermentable Oligo-, Di- and Mono-saccharides and Polyols-which includes fructose, lactose, oligosaccharides, polyols, and sugar alcohols (polyols, such as sorbitol, mannitol, xylitol and maltitol)), and dietary polyphenols may increase the abundance of *A. muciniphila* in healthy humans or animals (35). In addition, prebiotics can reverse the reduction of *A. muciniphila* abundance due to high-sugar or high-fat diets (36, 37). Studies have shown that the abundance of *A. muciniphila* in the feces of DIO mice decreased from 10^9^/g to 10^7^/g after being fed a high-fat diet (containing 60% fat) for 8 weeks; however, supplementation with low-fructo-oligosaccharides (0.3 g/day, high-fat diet) for 8 weeks restored its abundance to the original level (36). Additionally, in ob/ob mice, after 5 weeks of oral administration of low-fructo-oligosaccharides at a dose of 0.3 g/day, the abundance of *A. muciniphila* in the intestines increased by more than 80 times, along with a significant increase in the abundance of *Bifidobacterium* spp. and the *E. rectale/C. coccoides* group (38). Furthermore, a FODMAP diet has been found to alleviate Crohn’s Disease. A single-blind, randomized, crossover trial compared the effects of low (3.05%) and high intake of FODMAP (“typical Australian diet” containing 23.7% FODMAP) on Crohn’s Disease. The results showed that the low FODMAP diet reduced the total bacterial abundance, while A typical Australian diet increased the relative abundance of the butyrate-producing *Clostridium cluster XIVa* and the mucus-associated *A. muciniphila*, and reduced the torque of Fusobacterium (39). Moreover, dietary polyphenols play a role in regulating the gut microbiota. Compared with the control group, a high-fat diet containing 1% Concord grape polyphenols significantly increased the abundance of *A. muciniphila* in the intestines of C57BL/6J mice and reduced the ratio of *Firmicutes* to *Bacteroidetes* (40). Anthocyanin supplements significantly increased the β-diversity of the gut microbiota and the abundance of Bacteroidetes (41). Mustard polyphenols also reduced the ratio of *Firmicutes* to *Bacteroidetes* and increased the abundance of *Lactobacillaceae*, *A. muciniphila*, and *Blautia* (42). Orange polyphenols promoted the increase of *Bifidobacterium* and *Lactobacillus* (43). The third influencing factor is disease. As an important tissue in direct contacting with the external environment, disruption of the structure and abnormal composition of intestinal microorganisms can trigger disease, and in turn, disease may disrupt its healthy microecology. Most diseases, including metabolic disorders, neurodegenerative diseases, immune system disorders, cancer and intestinal inflammation, have a negative association with *A. muciniphila* (44). The final factor to consider is antibiotics. These drugs are used for preventing or treating bacterial infections and can be categorized based on their molecular structures into groups like β-lactams, penicillins, macrolides, tetracyclines, aminoglycosides, among others. Most antibiotics possess broad-spectrum activity. In a double-blind human trial, significant changes in the composition of intestinal microbiota were observed on days 7, 12, 17, and 27 following a 7-day eradication therapy comprising amoxicillin 500 mg qid, metronidazole 400 mg tid, and lansoprazole 30 mg bid. Comparing fecal microbiota abundance on day 1 and day 27 post-treatment revealed a decrease in *Bacteroides* genus and total *lactobacilli*, while the abundance of *Bifidobacterium* genus, *Enterobacteriaceae*, and *Enterococcus/Streptococcus* genus showed an increasing trend (45). The impact of antibiotics on gut microbiota is influenced by several factors, including spectrum of activity, pharmacokinetics, dosage, route of administration, and intestinal concentration (46). Additionally, a novel antibiotic, OPS-2071 (a quinolone class drug), demonstrates high antibacterial activity against other intestinal bacterial strains but notably lower activity against *A. muciniphila*. OPS-2071 significantly increases the colonization rate of *A. muciniphila* and the mucin content in the feces of healthy rats (47). Similar experimental results were observed in a human study using vancomycin treatment, where *A. muciniphila* significantly colonized the intestine after broad-spectrum antibiotic treatment (8), although the underlying mechanisms remain unclear. Additionally, geography, lifestyle, baseline levels, and the host genotype all impact its abundance.

## 3 The utility of *A. muciniphila* in metabolic diseases

The disruption of gut homeostasis in metabolic diseases is closely linked to intestinal immune function. Acting as a physiological barrier, the intestine prevents harmful substances from infiltrating the body. The intestinal barrier comprises biological, physical, chemical, and immune components (48). Among them, biological barrier refers to the fact that probiotic bacteria occupy intestinal space and compete for nutrients and survival resources, thereby limiting the growth and reproduction of pathogenic microorganisms (49). The composition and structure of the microbiota influence the host immune system by regulating the intestinal mucosal barrier, producing metabolic products, and modulating immune responses (50). Concurrently, the host immune system sustains intestinal microbial balance by recognizing and eliminating harmful microbes and controlling immune reactions (51). The intricate interplay between microbes and hosts is essential for preserving intestinal health, warding off infections, and modulating immune responses.

Metabolic disease is a comprehensive disease based on obesity. According to the International Diabetes Federation, the metabolic disease is diagnosed when central obesity is accompanied by any two of the following conditions: ① Triglyceride levels of 150 mg/dL (1.7 mmol/L) or more; ② High-density cholesterol decreasing to 40 mg/dL (1.3 mmol/L) in men and 50 mg/dL (1.29 mmol/L) in women; ③ Elevated blood pressure to >130 mm Hg systolic or >85 mm Hg diastolic, or a diagnosis of hypertension. ④ Fasting blood glucose exceeding 100 mg/dL (5.6 mmol/L) or diagnosed with T2DM. This criterion has been widely accepted and revised. Further analysis of the intestinal microbiota of patients with metabolic diseases (obesity, T2DM, CVD and NAFLD) indicated that the abundance of *A. muciniphila* microbiota was reduced, suggesting that there must be a direct or indirect association between the two. However, the exact mechanism underlying this association is not fully understood.

### 3.1 *A. muciniphila* and obesity

In recent years, people’s dietary needs have expanded beyond subsistence level. Obesity has become another significant consequence of the affluence of material possessions. It is now one of the leading causes of health risks and a risk factor for many diseases, including T2DM, NAFLD, and CVD (52, 53), making obesity one of the most important public health problems of the 21st century (54). The regulation of intestinal microbiota has garnered widespread interest as an effective strategy to prevent or treat obesity. *A. muciniphila*, the only Verrucomicrobia (phylum) genus that can be cultured *in vitro*, has been repeatedly mentioned in the context of obesity. The objective regulations that exist between the two has been gradually revealed. In 2010, it was observed for the first time that the abundance of *A. muciniphila* was lower in overweight pregnant women than in normal-weight pregnant women (55). Similar findings were observed in the stool samples of obese or overweight preschool children (56). Furthermore, a study analyzing fecal microbiota in 17 lean weight (BMI 19-24.99 kg/m^2^) and 15 obese women (BMI>30 kg/m^2^) using real-time fluorescence quantification PCR (qPCR) method revealed that *A. muciniphila* was more abundant in lean individuals than in obese ones (57). To further investigate the relationship between *A. muciniphila* and obesity, researchers successfully induced an obese mouse model with a high-fat diet and collected cecal feces. Analysis of feces by qPCR showed a 100-fold decrease in the population of *A. muciniphila* in the obese model group compared to lean littermates. The ob/ob mice are homozygous Lepob mutant mice with an invisible gene on chromosome 6 that causes obesity and advanced diabetes. Similarly, analysis of their intestinal feces showed a 3300-fold reduction in *A. muciniphila* population in ob/ob mice compared to lean littermates (36). The substantial changes in *A. muciniphila* genus abundance indicate a possible association between *A. muciniphila* and obesity. However, a few studies contradicted the above findings. For instance, *A. muciniphila* was more abundant in obese than in normal-weight children (58). To comprehensively elaborate the regularity between the two, substantial human and animal studies were conducted, which conclusively demonstrated that *A. muciniphila* abundance was reduced in obese individuals or animals (**Table 1**).

**Table 1:** The correlation between *A. muciniphila* abundance levels and metabolic diseases.

Research subjects	Condition	Study grouping	Sample collection and testing methods	Variation in abundance of *A. muciniphila*	Ref.
Human	Obesity	30 volunteers from Colombians	Feces 16S rRNA sequencing	↓: decreased in volunteers with high BMI.	(59)
Mice	Diet-induced obesity	N = 6, chow diet N = 6, high-fat diet (HFD)	Cecal sample qPCR	↓: progressively declined in HFD group	(60)
Mice	HFD induced obesity	N = 7, chow die N = 7, HFD	Feces qPCR	↓: lower in HFD group.	(61)
Mice	HFD induced obesity	N = 6, normal diet N = 6, HFD	Feces 16S rRNA sequencing and qPCR	↓: lower in HFD group.	(62)
Human	Obesity	N = 50, normal weight N = 50, obesity	Feces qPCR	↓: significantly decrease in obese group	(63)
Mice	Diet-induced obesity	N = 12, low fat diet N = 12, HFD	Cecal sample 16S rRNA sequencing	↓: lower in HFD fed mice.	(64)
Human	Obesity and T2DM	N = 151, T2DM patients N = 50, healthy controls	Feces 16S rRNA sequencing	↓: decreased in T2DM patients	(65)
Human	Lean T2DM	N = 22, control N = 52, abdominal obesity N = 22, T2DM N= 56, T2DM and abdominal obesity	Feces Metagenomic	↓: decreased in lean T2DM patients	(66)
Mice	NASH	N = 6, control N = 6, methionine- choline-deficient (MCD) diet	Feces 16S rRNA sequencing	↓: decreased in MCD group	(67)
Mice	NAFLD	N = 6, normal chow N = 6, HFD N = 6, chow + betaine N=6, HFD + betaine	Feces 16S rRNA sequencing	↓: decreased in the lean HFD group	(68)

"↓" represents a decrease in the abundance of *A. muciniphila*.

Based on the phenomenon of decreased *A. muciniphila* abundance due to obesity, researchers hypothesized that supplemental *A. muciniphila* could alleviate obesity or pathological features resulting from obesity. Amandine Everard et al. in 2013 investigated the beneficial effects of *A. muciniphila* on high-fat diet-induced obese mice (live and heat- killed at 121°C for 15 min). The results demonstrated that live *A. muciniphila* could alleviate fat-mass gain, insulin resistance, adipose tissue inflammation, and metabolic endotoxemia in obese mice, whereas heat-killed *A. muciniphila* had no such effects (36). In addition, numerous animal experiments have discovered that *A. muciniphila* supplementation also has physiological activities including reducing body weight, promoting metabolism, and enhancing intestinal barrier (**Table 2**), However, *A. muciniphila* exacerbated intestinal inflammation in salmonella-infected gnotobiotic mice, which is a negative effect (78). Studies have shown that *A. muciniphila* extracellular vesicles reduce intestinal permeability (72). *A. muciniphila* also exacerbates depletion of the mucus layer by consuming mucin, which in turn leads to thinning of the mucus layer and increased inflammation. The application of *A. muciniphila* in humans has been relatively slow due to the uncertainty of its functional activity and the lack of complete understanding of its mechanism. Hubert Plovier et al. first introduced pasteurized (70°C, 30 min) *A. muciniphila* and unexpectedly observed that pasteurization enhanced the functional activity of *A. muciniphila*, which reduced fat-gain, insulin resistance and dyslipidemia in obese mice (6). This surprising discovery expands the potential of *A. muciniphila* as a next-generation probiotic for food applications. In a subsequent randomized, double-blind, placebo-controlled exploratory study, overweight or obese volunteers were supplemented with 10^10^ live or pasteurized *A. muciniphila* daily for three months. This study provided groundbreaking evidence that *A. muciniphila* is safe and tolerable. In addition, pasteurized *A. muciniphila* still retains the capacity to lower total plasma cholesterol and reduce body weight, body fat and hip circumference in humans (73). Toxicological experiments have also confirmed the safety of pasteurized *A. muciniphila* as a food ingredient (79). Although current research results are promising, there are limitations in the study subjects. Therefore, further studies are required to expand the scope of subjects and demonstrate the relationship between *A. muciniphila* supplementation and improving obesity metabolic parameters.

**Table 2:** Beneficial effects of *A. muciniphila* and its derivatives supplementation on metabolic diseases.

Viability of *A. muciniphila*	Disease	Study grouping	Daily dose and period of administration	*A. muciniphila* validity conclusions	Ref.
*A. muciniphila*	HFD-fed obese mice	N = 6, HFD N = 6, HFD + AKK	① HFD group: PBS ② HFD + AKK group:4.0× 10^8^ CFU AKK ③ 6 weeks of oral administration	↑glucose tolerance ↓adipose tissue inflammation	(69)
*A.muciniphila*, Heat-killed *A. muciniphila*	High-fat, high- sucrose (HF/HS) diet induced obese mice	N = 5, HF/HS + AKK N = 5, HF/HS + H-K-AKK	① HF/HS+AKK group: 1.44 × 10^9^ CFU/0.2 ml AKK; ② HF/HS+ H-K-AKK group: 1.44 × 10^9^ CFU/0.2 ml H-K-AKK; ③ 5 weeks of oral administration.	↓body weight; ↓total body fat; ↑metabolic parameters.	(70)
*A. muciniphila*	HFD-fed obese mice	N = 4-5, HFD N = 4-5, HFD + AKK mucin (+) N = 4-5, HFD + AKK mucin (-)	① HFD group: 0.15 ml sterile anaerobic PBS (containing 25% vol/vol glycerol); ② HFD +AKK mucin (+) group: 1 × 10^8^ CFU/day (AKK grown on mucus-based medium); ③ HFD +AKK mucin (-) group: 1 × 10^8^ CFU/day (AKK grown on mucus-depleted medium); ④ 4 weeks of oral administration.	HFD +AKK mucin (-) group was more efficiently than HFD +AKK mucin (+) group in aspect of: ↓obesity; ↑barrier integrity.	(71)
AmEVs	HFD induced a diabetic phenotype	N = 5–7, ND N = 5–7, NCD + AmEVs N =5–7, HFD N = 5–7, HFD + AmEVs	① ND and HFD group: PBS; ② NCD + AmEVs and HFD + AmEVs group: 10 μg per mice AmEVs ③ 2 weeks of oral administration.	AmEVs: ↑: glucose tolerance; ↑: body weight; ↑: intestinal barrier function.	(72)
*A.muciniphila*; Pasteurized *A.muciniphila*	HFD induced a diabetic phenotype	N=11, placebo N=12, pasteurized AKK N=9, AKK	① placebo group: placebo; ② pasteurized AKK group: 1*10^10^ CFU/day/volunteer; ③ 3 months of oral administration.	↓: relevant blood markers of liver dysfunction and inflammation.	(73)
*A.muciniphila*; Heat killed *A. muciniphila*	Atherosclerosis	N=8-10, NCD N=8-10, Western diet (WD) N=8-10, WD + AKK N=8-10, WD + heat- killed AKK	① NCD and WD group: 200 µl PBS; ② WD+ AKK and WD+ heat killed AKK group: 5×10^9^ CFU/200 µl; ③ 8 weeks of oral administration.	AKK reversed Western diet–induced exacerbation of atherosclerotic lesion formation without affecting hypercholesterolemia.	(74)
*A.muciniphila*	Fatty Liver Disease	N=5, ND + PBS N=5, ND + AKK N=5, HFD + PBS N=5, HFD + AKK	① ND+PBS and HFD+ PBS group: PBS; ② ND+ AKK and HFD+ AKK group: 10^8^ to 10^9^ CFU/ml; ③ 10 weeks of oral administration.	↓: lowered serum triglyceride and alanine aminotransferase levels in obese mice; ↓: the expression of SREBP (regulator of TG synthesis in liver tissue).	(75)
*A.muciniphila*	NASH	N=5-8, High-fat and high-cholesterol (HFC) N=5-8, HFC + AKK	① HFC group: HFC diet+ 200 µl PBS; ② HFC + AKK group: HFC diet + 1 × 10^8^ CFU/ml/200 µl, ③ 6 weeks of oral administration.	↓: Hepatic steatosis/inflammatory ↓: serum ALT/AST/ALP ↓: hepatic genes expression related to steatosis and inflammation	(76)
*A.muciniphila*	NASH-induced cognitive damage	N=8, NC group N=8, HFHC + PBS N=8, HFHC + Lacticaseibacillus rhamnosus GG(LGG) N=8, HFHC + AKK	① ND group: normal chow; ②HFHC + PBS group: HFHC +100 µl PBS; ③HFHC + LGG group: HFHC + 1*10^9^ CFU/100 μl LGG; ④HFHC + AKK group: HFHC + 1*10^9^ CFU/100 μl AKK; ⑤4 weeks of oral administration.	↓: HFHC-induced cognitive dysfunction (including impaired spatial working memory and novel object recognition)	(77)

"↑" means promotion or enhancement; "↓" means reduction or alleviation.

As the effects of *A. muciniphila* in alleviating obesity continue to be demonstrated, its underlying mechanisms are being explored. Obesity is a chronic metabolic disease caused by the excessive accumulation or abnormal distribution of organismal fat, mainly due to the imbalance between caloric intake and energy excretion of the body. When caloric intake exceeds energy excretion, excessive energy is stored as fat in adipocytes. Adipocytes are essential for fat synthesis and storage. Glycerol and fatty acids required for triglyceride synthesis are mainly provided by glucose metabolism. Glycerol is converted from dihydroxyacetone phosphate produced by glycolysis and fatty acids are synthesized from acetyl coenzyme A produced by the oxidative breakdown of glucose. Pasteurized *A. muciniphila* alleviates diet-induced obesity through increased energy expenditure and spontaneous physical activity. The energy expenditure is not associated with changes in thermogenic markers or white adipose tissue but with decreased expression of the lipid droplet-associated factor perilipin2 in brown and white fat. In addition, pasteurized *A. muciniphila* decreases carbohydrate absorption, increasing energy excretion in the feces (80). The treatment of 3T3-L1 cells with *A. muciniphila* cell lysate effectively diminished lipid accumulation and down-regulated mRNA expression of adipogenesis-associated proteins. *A. muciniphila* upregulates the expression of SERPINA3G in adipocytes and inhibits adipogenesis (81). Currently, the study of the molecular mechanism of *A. muciniphila* in alleviating obesity is still in the initial stage, additional studies are needed to further elucidate it.

### 3.2 *A. muciniphila* and T2DM

Diabetes mellitus is a chronic metabolic disease caused by the deficiency in insulin secretion or the reduction in the body’s ability to utilize insulin. T2DM accounts for approximately 90% of diabetes mellitus cases worldwide. According to the 10th edition of the International Diabetes Federation, in 2021 there were 537 million adults (20-79 years old) with diabetes and more than 4 million deaths per year were caused by diabetes. Studies have demonstrated dramatic differences in the structure and composition of the gut microbiota in T2DM patients compared with healthy individuals, including *A. muciniphila*. In 2013, a study was conducted on normal, pre-diabetic and diabetic individuals using 16S rRNA sequencing of stool samples to quantify *A. muciniphila*. The results showed that *A. muciniphila* abundance was significantly lower in the pre-diabetic and diabetic groups compared to the normal group (82). Another study was conducted on the stool samples of patients with short-, medium- and long-term T2DM. The results indicated that the abundance of *A. muciniphila* in medium- and the long-term patient was significantly lower than that of short-term patients. The above studies strongly indicated a correlation between the pathological process and *A. muciniphila* abundance, and the longer the disease duration, the lower the *A. muciniphila* abundance. However, there are a few studies that contradict the above conclusion. Marion Régnier et al. induced a T2DM mouse model with a high-fat and high-sugar diet, collected feces, and analyzed them using 16S rRNA sequencing combined with qPCR. The results showed that *A. muciniphila* was more abundant in T2DM mice compared to normal mice (83). Another study showed that *A. muciniphila* was more abundant in T2DM patients than in healthy individuals (84). The inconsistency of the findings prompted a large number of studies to be conducted, which eventually concluded that there is a negative correlation between the two (**Table 1**). This negative correlation property suggests that *A. muciniphila* supplementation may reverse the underlying physiological indices of T2DM. To test this hypothesis, researchers induced T2DM mice with a high-fat diet and gavaged them 2 x 10^8^ CFU/0.2 ml/day for four weeks. The results showed that *A. muciniphila* possessed the physiological activity to alleviate the elevated blood glucose induced by the high-fat diet (36). Meanwhile, Feifan Wu et al. reported that *A.muciniphila* could improve glucose tolerance, regulate intestinal microbiota destroyed by high-fat diets, promote acetate and propionate production, and enhance intestinal barrier function (22). Numerous subsequent studies confirmed these functional activities.

Obesity is a crucial factor in the pathogenesis of T2DM, as evidenced by studies conducted on animal models induced with high-fat diets combined with streptozotocin. The primary pathological mechanisms of T2DM have been identified as: ① Insufficient insulin secretion. Obesity often leads to dyslipidemia and chronically elevated levels of free fatty acids (FFAs). FFAs are essential to maintain the function of islet cell (85). However, a high level of FFA stimulation can lead to a decline in islet cell function. In one study, when isolated islets were exposed to high levels of FFA, insulin secretion significantly decreased over time and eventually ceased, leading to non-insulin secretion (86). High levels of FFA oxidation in islet cells reduced the expression of glucose transporter receptor 2, glucokinase and insulin genes, which directly affected insulin synthesis and secretion (87). As the functional activity of *A. muciniphila* in modifying diabetes is further investigated (**Table 2**), its intrinsic molecular mechanisms are being revealed. *A. muciniphila* has the function of reducing FFA levels by alleviating obesity. In addition, *A. muciniphila* improved the structure and composition of the intestinal microbiota and promoted the production of short-chain fatty acid (88). Acetic acid, propionic acid and butyric acid targeted G protein-coupled receptors 41 and 43 on the surface of intestinal L cells, which activated downstream molecular signaling and ultimately promoted the secretion of glucagon-like peptide-1(GLP-1) (89). Furthermore, *A. muciniphila* can secrete glucagon-like peptide-1-inducing protein P9. P9 bound to intercellular adhesion molecule 2 on the surface of L cells and activated downstream molecular pathways to stimulate the secretion of GLP-1 (90). GLP-1 is transported to the pancreas through blood vessels, and binds to its corresponding receptors to induce insulin secretion, ultimately achieving hypoglycemic effect. ② Insulin resistance. Obesity disrupts intestinal microbiota and elevated levels of lipopolysaccharides (LPS). High levels of LPS penetrate the intestinal barrier and activate the NF-κB/MAPKs signaling pathway, resulting in chronic low-grade inflammation (91). As chronic low-grade inflammation is generated, serine kinases (JNK, IKK) are activated and induce phosphorylation of insulin receptors, preventing insulin from binding properly to its receptors (92), leading to insulin resistance. LPS crossing the intestinal barrier is a central cause of chronic low-grade inflammation and is a key step in developing insulin resistance. The intestinal barrier function is maintained by two main factors: the thickness of the mucus layer and the degree of tight junctions between intestinal epithelial cells. The mucus primarily comprises water, inorganic salts and mucin, and mucin is its main functional component. The most abundant protein in the mucin complex is MUC2. The degree of tight junctions between intestinal epithelial cells is determined by the proliferation capacity of intestinal epithelial cells and the expression level of tight junction proteins. It has been reported that *A. muciniphila* upregulates MUC2, BIRC3 and TNFAIP3 (BIRC3 and TNFAIP3 are anti-apoptotic genes of intestinal epithelial cells involved in their proliferation process) via ADP-heptose-dependent activation of the ALPK1/TIFA pathway, which maintains intestinal barrier function and reduce its permeability (93). This subsequently inhibits chronic low-grade inflammation induced by LPS and insulin resistance. In addition, *A. muciniphila* promotes the expression of the tight junction proteins ZO-1, Occludin, and Claudin 3 (94). The outer membrane protein Amuc_1100 from *A. muciniphila* (95), and its extracellular vesicle (72) can enhance intestinal barrier function. In conclusion, *A. muciniphila* and its derivatives alleviate T2DM by promoting insulin secretion and reducing insulin resistance.

Metformin, as a first-line treatment for type 2 diabetes, has a good safety profile, but its exact mechanism of action is currently unclear. While traditionally thought to lower blood sugar levels by activating the hepatic AMPK pathway, thereby reducing hepatic glucose output (96–101), recent findings suggest metformin may also influence intestinal pathways (102, 103). Metformin can rapidly change the composition and structure of the intestinal microbial flora, mainly by reducing the diversity of the bacterial flora in mice and promoting the abundance of *A. muciniphila* and various short-chain fatty acid-producing microbial flora in the human intestine, including *Butyrivibrio*, *Bifidobacterium bifidum*, *Megasphaera*, and an operational taxonomic unit of *Prevotella* (104, 105). Metformin’s positive regulation of intestinal flora is beneficial to increase the production of short-chain fatty acids. Short-chain fatty acids can promote the proliferation of intestinal mucosal epithelial cells and enhance the expression of tight junction proteins, thereby improving intestinal barrier function (106). Strengthening the intestinal barrier effectively suppresses inflammation triggered by lipopolysaccharide penetration. Additionally, short-chain fatty acids bind to G-protein-coupled receptors, prompting intestinal L cells to release PYY and GLP-1. PYY curbs appetite and slows gastrointestinal motility, aiding in obesity management (107), while GLP-1 stimulates insulin release by binding to pancreatic islet B cell receptors, thereby regulating blood sugar levels (108). In summary, metformin’s modulation of intestinal flora is intricately linked to its anti-inflammatory, anti-obesity, and hypoglycemic effects.

During the modulation of gut microbiota, the impact of metformin on the levels of *A. muciniphila* is consistently highlighted. C57BL/6 mice were fed a high-fat diet (60% fat) for 28 weeks to induce metabolic disorders (obesity and T2D), and then treated with metformin at a dose of 300 mg/kg/day for 10 weeks to observe the effect of metformin on the intestinal microbiota. The findings indicated a notable increase in the abundance of *A. muciniphila* and *Clostridium cocleatum* in mice receiving metformin post high-fat diet (109). In an experiment using metformin to treat ulcerative colitis, the ability of metformin to increase the relative abundance of *Lactobacillus* and *Akkermansia*, reduce *Clostridium erysipelvis* at the genus level, and alter the composition of the intestinal microbiota was similarly demonstrated (110). Presently, there’s no evidence to suggest direct stimulation of *A. muciniphila* production by metformin; however, studies have noted a significant rise in goblet cell count in mouse intestines post-metformin treatment. This increase in mucin secretion by goblet cells directly correlates with the elevated abundance of *A. muciniphila* (69). In summary, metformin achieves the effect of alleviating T2DM by regulating the abundance of intes tinal flora, especially *A. muciniphila*.

### 3.3 *A. muciniphila* and CVD

CVD is the leading cause of mortality worldwide (111). According to the World Health Organization, approximately 17.9 million people died from CVD in 2019, accounting for 32% of all deaths worldwide. There are significant differences between the intestinal microbiota of CVD patients and healthy individuals. A survey of patients with major adverse cardiovascular and cerebrovascular events (MACCE) showed that *Eubacterium eligens*, *A. muciniphila*, *Prevotella stercorea*, and *Eubacterium rectale* were less abundant in MACCE patients than in the no-MACCE group (112) (**Table 1**). Notably the amount of *A. muciniphila* in the intestine of abdominal aortic aneurysms patients or mice was nearly depleted. This predicts that *A. muciniphila* may play a crucial role in their pathological process. *A. muciniphila* supplementation has been found to reverse Western diet-induced atherosclerosis, which is a major pathological process in CVD. In addition, *A. muciniphila* (2 × 10^8^ CFU/180µl) improved cardiovascular disease in mouse models by altering the intestinal microbiota and immune system (113). *A. muciniphila* had also been demonstrated to improve cold-associated atrial fibrillation by inhibiting the formation trimethylamine (TMA)/trimethylamine N-oxide (TMAO) in a mouse model of cold-associated atrial fibrillation (114). Currently, the physiological activity of *A. muciniphila* for CVD has been validated to a certain extent. However, relevant studies and clinical data are still relatively scarce. Therefore, further studies need to be conducted.

Atherosclerosis, the leading cause of CVD (115), is manifested as atheromatous plaque material composed of lipid or fibrous substances in the entire aorta and arterial roots. The main mechanism of atherosclerosis: ① With the improvement of people’s living standard, high-fat diet gradually becomes people’s daily diet pattern. Long-term consumption of high-energy diets can induce obesity and cause abnormalities in the body’s lipid metabolism. As a result, high levels of oxidized low-density lipoprotein cholesterol in the plasma are not transferred out of the plasma in time to be deposited in the inner wall of the arterial vessels and trigger inflammation, resulting in the gradual formation of atheromatous deposits in the lumen of the arteries. Over time, this deposit eventually becomes fibrotic, resulting in narrowing of the arterial lumen, causing a loss of wall elasticity and plaque rupture. The ruptured plaques circulate with the blood, easily blocking the blood vessels and causing thrombosis, eventually leading to the occurrence of adverse CVD, including myocardial infarction and angina pectoris, which is fatal for human health and life (116). Oral administration of *A. muciniphila* can reverse weight gain and abnormal lipid metabolism caused by a high-fat diet and reduce plasma cholesterol levels. In addition, *A. muciniphila* can enhance intestinal barrier function, which effectively inhibits LPS-induced inflammation and indirectly prevents CVD. ② Choline, phosphatidylcholine, and L-carnitine are abundant in red meat, shellfish, eggs, and fish. These three substances can be metabolized by intestinal microbiota to TMA, a CVD biomarker. TMO absorbed by the organism is further converted to TMAO by hepatic flavin monooxygenase 3 (FMO3) in the liver. Pasteurized *A. muciniphila* can counteract the increase in FMO3 levels induced by high-fat diet, suggesting the possibility of *A. muciniphila* intervention in TMAO production. Furthermore, supplementing obese mice with *A. muciniphila* could promote the excretion of TMA and TMAO through urine, thereby reducing their levels in plasma. In conclusion, *A. muciniphila* can effectively inhibit the occurrence of CVD through different pathways.

### 3.4 *A. muciniphila* and NAFLD

NAFLD is a pathological syndrome characterized by excessive deposition of lipids in liver cells caused by factors other than alcohol and clear liver damage factors (primarily drugs, viral infections, and autoimmunity) (117). It has a high prevalence and incidence in many countries and can be divided into three categories based on its pathological process: simple fatty liver, non-alcoholic steatohepatitis (NASH), and liver cirrhosis. Intestinal microbiota has gained considerable attention as a potential therapeutic target for NAFLD. Studies showed that abundance of *A. muciniphila* was significantly reduced in gut of NASH and NAFLD patients (118) (**Table 2**). A study conducted on 46 NASH patients and 38 healthy controls also confirmed the above findings based on qPCR results of their stool samples (119). In addition, using saccharin/sucalose to induce the NAFLD mouse model resulted in a significant decrease in the abundance of *A. muciniphila* (120). However, while most studies suggest a negative relationship between the two, some findings contradict it. GV Moreira et al. induced a mouse model with NAFLD phenotype using a high-fat diet and observed an increase in the abundance of *A. muciniphila* in the intestinal tract of the model (4). The inconsistency of conclusions suggests the need for further exploration of the relationship between the two. The significant reduction in the abundance of *A. muciniphila* in NAFLD patients suggests its potential role in alleviating NAFLD. Yong Rao et al. used high cholesterol and high fat to induce mice and administered *A. muciniphila* at a dose of 1x10^8^ CFU/mL/day for 6 weeks. The results showed that it effectively reversed NAFLD in the liver, including hepatic steatosis, inflammation, and liver injury (121). At the same time, *A. muciniphila* supplementation can significantly reduce liver fat and the risk of NAFLD. Currently, no clinical data support this conclusion, and further verification is needed.

Abnormal accumulation of fat in liver cells is a prerequisite for the formation of NAFLD, which is caused by disorders of liver lipid metabolism. The homeostasis of hepatocyte lipid metabolism mainly depends on the dynamic balance of fatty acid uptake, fatty acid synthesis, and lipolysis processes. Glycerol and fatty acids are the raw materials required for synthesizing triglycerides. Glycerol is converted from dihydroxyacetone phosphate produced by glucose in the intestine. Fatty acids are primarily derived from three sources: ① Triglycerides in adipose tissue are decomposed into FFA, and some FFA participate in fat synthesis in the liver. The fat synthesized through this pathway accounts for 59% of the total fat in the liver. This process is primarily associated with regulating insulin signaling. Adipose tissue will abnormally decompose excess FFAs into the liver after insulin resistance, promoting NAFLD development. ② Glucose and fructose from carbohydrates in the food are digested and absorbed by the small intestine to form fatty acids through the adipogenesis pathway. The fatty acids are then transported to the liver for fat synthesis, which accounts for 26% of the total fat in the liver. ③ Excessive intake of saturated fatty acids in the diet will also affect the synthesis of liver fat, which accounts for 15% of the total fat in the liver. *A. muciniphila* can alleviate the abnormal decomposition of adipose tissue by reducing insulin resistance (**Table 2**). Additionally, supplementing pasteurized *A. muciniphila* reduces carbohydrate absorption, thereby reducing the production of glucose and fructose and effectively inhibiting the production of FFAs. The formation of NAFLD is also related to the timely consumption of fatty acids or fats. Supplementation with *A. muciniphila* increased the levels of L-aspartate in the gut-liver axis. L-Aspartate stimulated lipid oxidation and released energy by activating the hepatic LKB1-AMPK axis, thereby reducing liver fat accumulation. In conclusion, *A. muciniphila* can reduce the accumulation of fatty acids or fat in the liver through a variety of mechanisms, thereby alleviating NAFLD.

## 4 Conclusion

**Figure 2:**
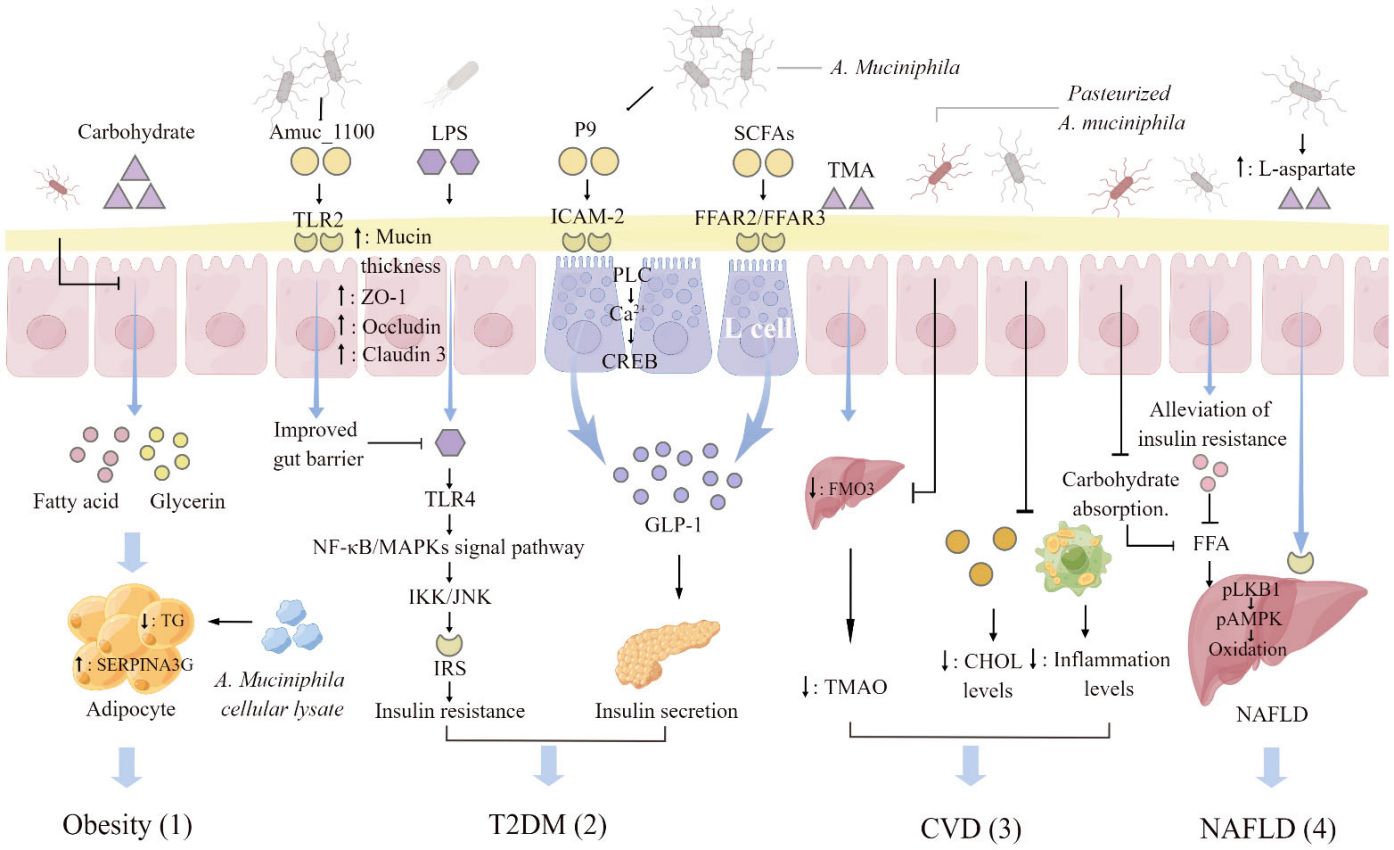
*A. muciniphila* regulating mechanisms associated with metabolic diseases. (1) Pasteurized *A. muciniphila* reduces glycerol and fatty acid levels and triglyceride (TG) production through inhibiting carbohydrate absorption. *A. muciniphila* cellular lysate upregulates serine protease inhibitor peptidase inhibitor clade 3G (SERPINA3G) expression in adipocytes and inhibits adipogenesis. (2) Activation of toll-like receptors 2 (TLR2) by Amuc _1100, an outer membrane protein of *A. muciniphila*, increases the thickness of the mucus layer and promotes the expression of tight junction proteins ZO-1, Occludin, and Claudin3. The enhancement of intestinal barrier function will hinder the insulin resistance induced by the activation of NF-KB/MAPKs signaling pathway caused by the combination of lipopolysaccharide (LPS) penetration and toll-like receptors 4 (TLR4). The glucagon-like protein P9 produced by *A. muciniphila* binds to the intercellular adhesion molecule 2 (ICAM-2) on the surface of L cells and activates phospholipase C (PLC), intracellular Ca^2+^ signaling, and CREB. P9 is involved in the secretion of GLP-1. Short- chain fatty acids (SCFAs) increased by *A. muciniphila* supplementation interact with free fatty acid receptor (FFAR) 2 and 3 on the surface of L cells to stimulate the secretion of GLP-1. (3) Pasteurized *A. muciniphila* inhibits the conversion of trimethylamine (TMA) to trimethylamine oxide (TMAO) by reducing the expression of hepatic flavin monooxygenase 3 (FMO3), and lowers the ratio of TAM/TAMO in plasma. *A. muciniphila* reduces the organism cholesterol (CHOL) and inflammation levels, indirectly reducing the risk of atherosclerosis and cardiovascular disease (CVD). (4) *A. muciniphila* indirectly decreases liver fat by regulating the level of free fatty acids (FFAs) in plasma. Supplementation with *A. muciniphila* increased the levels of L-aspartate in the gut-hepatic axis. L-Aspartate stimulate lipid oxidation and released energy by activating the hepatic LKB1-AMPK axis, thereby reducing liver fat accumulation. The figure was created using Figdraw.

The strategy of using gut microbiota to target the improvement of metabolic diseases has been validated, based on the amount of accumulated experimental results and clinical data. In this review, obesity, T2DM, CVD and NAFLD were studied as typical representatives of metabolic diseases and elaborates the intricate relationship between them and *A. muciniphila* (**Figure 2**). *A. muciniphila* is a potential candidate strain for “next-generation probiotics” and is frequently mentioned in the pathological process of metabolic diseases. The abundance of *A. muciniphila* in the gut has a significant negative correlation with metabolic diseases, and variations in its abundance are recognized as a biomarker for the occurrence of metabolic diseases. Identifying the specific molecular mechanism or mode of action by which *A. muciniphila* alleviates metabolic syndrome is critical. Some common pathways have been identified in obesity. Pasteurized *A. muciniphila* reduces energy intake by reducing carbohydrate absorption and increases fecal energy excretion. *A. muciniphila* lysate upregulates the expression of SERPINA3G in adipocytes to inhibit adipogenesis. Obesity is the underlying cause of metabolic diseases. Therefore, when obesity is reduced, certain physiological indicators of the body will also change, including the improvement of intestinal barrier function, the correction of abnormal lipid metabolism, the reduction of blood cholesterol level, and the decrease of blood FFAs content, which directly reduces the risk of T2DM, CVD and NAFLD. In addition, *A. muciniphila* and its derivatives can specifically inhibit the onset of these three diseases. *A. muciniphila*-derived protein P9 binds to intercellular adhesion molecule 2 in intestinal L cells and promotes the secretion of GLP-1. P9 finally achieves the effect of indirect stimulation of insulin secretion. Pasteurized *A. muciniphila* reduces the ratio of TAM/TAMO in plasma and indirectly inhibits CVD. *A. muciniphila* activates the hepatic LKB1-AMPK axis by increasing the level of L-aspartic acid in the gut-liver axis and increases lipid oxidation and energy release. Thereby, it reduces fat accumulation in the liver and alleviates NAFLD. This review elaborates the probiotic effect of *A. muciniphila* on metabolic diseases and provides a theoretical basis for the clinical application of *A. muciniphila* as a new functional strain.

## 5 Prospects and future challenges

*A. muciniphila* shows promise in improving metabolic diseases, with its abundance often inversely related to such disorders in both animal and human studies. However, the understanding of the specific biomolecules through which *A. muciniphila* influences metabolic diseases remains incomplete, a crucial aspect for its practical application. Currently, only a limited number of products containing heat-sterilized *A. muciniphila* are authorized for sale in Europe, facing significant marketization challenges. The main barriers to widespread use in clinical or food industries include: (1) Insufficient clinical data availability. (2) Need for further safety verification. (3) Challenges in cultivating *A. muciniphila* at high densities due to its anaerobic nature, leading to uncertainties in production feasibility while maintaining strain activity. While some clinical studies have demonstrated the probiotic effects of *A. muciniphila* and its safety in humans, limitations persist in terms of sample size, diversity, and demographics. Future research should focus on evaluating the physiological effects and safety of *A. muciniphila* on a larger scale, covering multiple dimensions comprehensively.

## Author contributions

HN: Writing – original draft, Writing – review & editing. XX: Writing – review & editing. MZ: Writing – review & editing. DZ: Writing – review & editing. ZX: Writing – review & editing. TW: Writing – review & editing. RC: Writing – review & editing. DC: Writing – review & editing. FL: Writing – review & editing.
